# Treatment of Chronic Anterior Shoulder Dislocation by Coracoid Osteotomy with or without Bristow–Latarjet Procedure

**DOI:** 10.1111/os.12776

**Published:** 2020-09-25

**Authors:** Yong‐liang Yang, Qing‐hu Li, Qi Zhang, Hong‐lei Jia, Bo‐min Wang, Jin‐lei Dong, Dong‐sheng Zhou, Xiao‐mei Wang, Lian‐xin Li

**Affiliations:** ^1^ Department of Traumatic Orthopedics Shandong Provincial Hospital affiliated to Shandong First Medical University Jinan China; ^2^ Department of surgery The Third People's Hospital of Lixia County Jinan China; ^3^ Nuclear Medicine Department Jinan Central Hospital Affiliated to Shandong University Jinan China

**Keywords:** Bristow, Chronic anterior shoulder dislocation, Coracoid osteotomy, Latarjet, Surgical treatment

## Abstract

**Objective:**

To investigate the clinical efficacy and outcomes of the coracoid osteotomy with or without Bristow–Latarjet procedures in the treatment of chronic anterior shoulder dislocation (CASD).

**Methods:**

Between January 2013 and January 2019, 20 shoulders of 18 patients who were diagnosed with chronic anterior dislocation and underwent open reduction in our trauma center were retrospectively studied. Open coracoid osteotomy with Bristow–Latarjet procedures were performed on 16 shoulders and open coracoid osteotomy without Bristow–Latarjet procedures were performed on four shoulders. Open coracoid osteotomy with or without Bristow–Latarjet procedures were chosen on the basis of the stability of the shoulder after reduction. Outcomes were assessed preoperatively and postoperatively with the visual analog scale (VAS) for pain, the American Shoulder and Elbow Surgeons (ASES) score, the University of California Los Angeles (UCLA) shoulder rating scale, and the range of motion (ROM) for shoulder activity.

**Results:**

There were three males and 15 females with an average age of 60.94 ± 2.69 years. The time between dislocation and treatment ranged from 21 to 240 days with an average of 73.3 ± 14.4 days. All patients were available for a mean follow‐up of 15.2 ± 4.3 months. No procedure‐related death or incision‐related superficial or deep tissue infection was identified in all cases. No iatrogenic neurovascular injuries or fractures were found in this study. At the time of 12 months follow‐up, the range of motion and the shoulder functional evaluation (VAS [*P* < 0.001], ASES [*P* < 0.001], and UCLA score [*P* < 0.001]) in patients who underwent Bristow–Latarjet procedures were significantly improved. Subluxation after surgical procedure was found and confirmed in one patient and this patient refused to undergo revision surgery. According to the Samilson and Prieto classification system, 16 shoulders were assessed as grade 0, three shoulders were grade 1, one shoulder was grade 2.

**Conclusions:**

Coracoid osteotomy with or without Bristow–Latarjet procedure yielded an acceptable clinical result in this study. This method has the advantages of enlarging the exposure of surgical field, assisting reduction of shoulder, and convenient conversion to Bristow–Latarjet procedure. It is an efficient and reliable method for treatment of chronic anterior shoulder dislocation.

A 69‐year‐old woman diagnosed with right chronic anterior shoulder dislocation with large Hill–Sachs lesion. The latarjet procedure with remplissage technique was applied for this patient.

## Graphical abstract

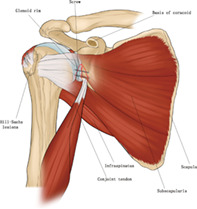

Treatment of chronic anterior shoulder dislocation with open coracoid osteotomy and latarjet procedure.

## Introduction

Shoulder dislocations account for almost 45% of all joint dislocations with more than 90% being the anterior subtype^1^. Most anterior dislocations have been manually reduced by the patient or by the surgeon in the emergency department. A chronic dislocation is defined as a case in which the diagnosis was missed for several days to weeks after initial dislocation^2^. The chronic anterior shoulder dislocation (CASD) is a rarer entity in orthopaedics, which often occurs in the elderly as a result of the patient's increasing age, weakness, and degeneration of the soft tissue surrounding the affected shoulder. The reasons of neglected shoulder dislocations in younger patients are almost always alcoholism, seizures, or multiple traumas^3, 4^. Though one study reported that the chronic anterior dislocation did not affect the function of the dislocated shoulder^5^, most patients with chronic anterior dislocation were unable to return to normal activities^6^, ^7^. Chronic anterior shoulder dislocation is commonly associated with several associated injuries, such as Hill–Sachs or Bankart lesions, glenoid fracture, massive glenoid bone loss, rotator cuff tears, or proximal humeral fractures^2^, ^8^.

Because of severe soft tissue contracture and imbalance as well as bone deficiency, chronic anterior shoulder dislocation is a difficult problem for both patients and clinicians. The optimal surgical technique for treatment of chronic anterior shoulder dislocation is still controversial^3^, ^6^, ^9^, ^10^. According to the literature reports, the treatment methods include closed reduction, open reduction and internal fixation with Kirschner wires, Bankart lesions repair with or without remplissage technique^9^, hemiarthroplasty^3^, reverse shoulder arthroplasty^10^, Bristow–Latarjet technique, coracoid transfer^6^, and bone grafting. Because of the complicated conditions of patients with chronic anterior shoulder dislocation, so far, no surgical technique has been unanimously approved by surgeons. However, the ability to restore the stability and activity of the shoulder is variable and the overall failure rate is fairly high^3^, ^11^, ^12^.

The coracoid bone block procedure was firstly described by Latarjet^13^ in 1954, then modified by Patte *et al* ^14^. and by Walch^15^ to increase the glenoid arc. Helfet described another similar coracoid transplantation procedure in 1958 and named it with his late mentor Bristow^16^. The Bristow–Latarjet procedure has been proven to be effective for the treatment of recurrent anterior shoulder dislocation with glenoid osseous defect more than 25%^17^, which might be used to treat chronic anterior shoulder dislocation^6^. Coracoid osteotomy with conjoined tendon, the critical process in the Bristow–Latarjet procedure, can not only assist to enlarge the surgical field of vision, but can also be transferred onto the neck of the scapula to prevent re‐dislocation. Some chronic anterior shoulder dislocation cases with glenoid fracture can be open‐reduced and remain stable by internal fixation with screws, with no need of Bristow–Latarjet procedure, the truncated coracoid process can be in situ, fixed with one or two screws. Therefore, the coracoid osteotomy is very important for chronic anterior shoulder dislocation.

The purpose of this study is as follows: (i) to summarize and observe the clinical features of chronic anterior shoulder dislocation; (ii) to report the effectiveness and clinical outcome of patients with chronic anterior shoulder dislocation treated with coracoid osteotomy with or without Bristow–Latarjet procedure; (iii) to explore the advantages of the coracoid osteotomy in the treatment of chronic anterior shoulder dislocation. The working hypothesis was that the coracoid osteotomy with or without Bristow–Latarjet procedure had the beneficial effect of convenience and safe early motion without the risk of re‐dislocation.

## Materials and Methods

### *Inclusion and Exclusion Criteria*

The inclusion criteria were as follows: (i) chronic anterior shoulder dislocations^6^ (for more than 3 weeks and irreducible on admission); (ii) the surgical technique was open reduction with coracoid osteotomy ± Bristow–Latarjet procedures; (iii) the major evaluation indicators included VAS, ASES score, UCLA score, ROM (3 weeks, 6 weeks, 3 months, 6 months, and 12 months after operation); (iv) a retrospective study.

The exclusion criteria were as follows: (i) the patients had congenital anterior shoulder dislocation; (ii) the patients had recurrent anterior shoulder dislocation; (iii) the patients had anterior shoulder dislocation with Charcot arthropathy; (iv) the patients had anterior shoulder dislocation less than 3 weeks; (v) the patients had severe medical comorbidities or inability to comply with postoperative rehabilitation.

### *General Information of the Cases*

**Fig 1:**
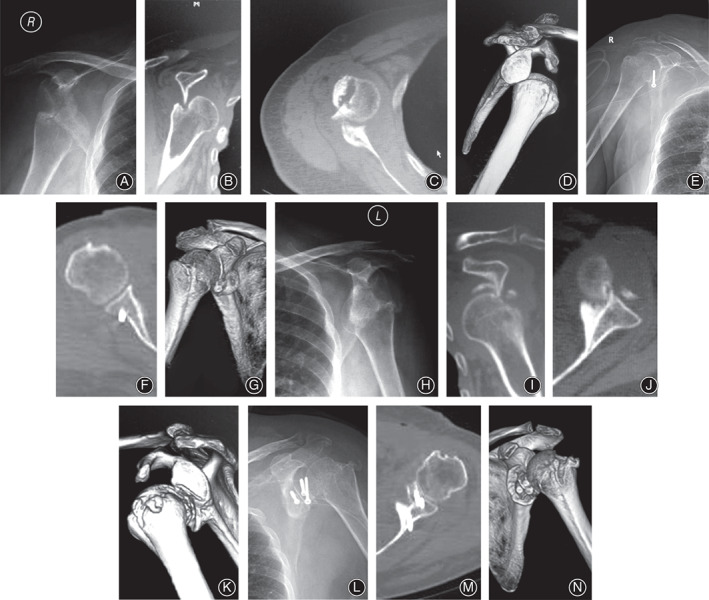
A 67 years old woman with bilateral chronic anterior dislocation, right anterior shoulder dislocation more than 2 months, left anterior shoulder dislocation more than 8 months (No. 17 patient). (A): Preoperative anteroposterior radiograph showing a chronic anterior dislocation. (B‐D): Preoperative CT scans showing chronic anterior dislocation with Hill‐Sachs lesion. Postoperative anteroposterior radiograph showing right shoulder reduction with Bristow procedure. (E‐G): Postoperative CT scans showed right shoulder was in restoration. (H): Preoperative anteroposterior radiograph showing a chronic anterior dislocation. (I‐K): Preoperative CT scans showing chronic anterior dislocation with Hill‐Sachs lesion and pseudoarthrosis formation. (L): Postoperative anteroposterior radiograph showing left shoulder reduction with Latarjet procedure and iliac graft block. (M, N): Postoperative CT scans showed left shoulder was in restoration and the location of screws was good.

Between January 2013 and January 2019, 20 patients with chronic anterior shoulder dislocation were referred to our hospital. Two female patients with chronic anterior shoulder dislocation more than 1 month were excluded because of Charcot arthropathy resulting from cervical syringomyelia. There were three males and 15 females with an average age of 60.94 ± 2.69 years (range, 30–75 years old). The mechanism of injury was fall in 12 patients, traffic accident injury in four patients, and wringer injury in one patient. Twelve dislocations involved the right arm and eight involved the left arm. Bilateral chronic anterior dislocations were found in two patients in this study, one patient's clinical data is shown in Fig. 1. All right shoulder dislocations involved the dominant limbs.

The time between dislocation and treatment ranged from 21 to 240 days with an average of 73.3 ± 14.4 days. The concomitant injuries included Bankart (± osseous) lesions in 12 shoulders, Hill–Sachs lesions in 12 shoulders, fractures of greater tuberosity in six shoulders, glenoid or scapula fractures in four shoulders, proximal humeral fractures in three shoulders, coracoid fractures in three shoulders, and rotator cuff tears in five shoulders. The patients' demographic data are listed in Table 1.

**TABLE 1:** The patients' demographic data

No.	Sex	Age (year)	Involved shoulder	Time of dislocation (days)	Concomitant injuries	Procedures
1	F	60	Left	40	GT fracture, RCT	Latarjet, RCT repair, ORIF
2	F	64	Right	60	Fractures of Proximal humerus and coracoid; osseous Bankart lesion, RCT	Bristow, ORIF, RCT repair
3	F	55	Right	150	Osseous Bankart, Hill‐Sachs lesion	Latarjet
4	F	69	Right	60	Bankart and Hill‐Sachs lesions	Latarjet, Remplissage
5	F	46	Right	60	Bilateral fractures of GT, RCTs, Hill‐Sachs lesions	Latarjet, ORIF, RCT Repair
			Left	60		Bristow, RCT Repair
6	M	30	Right	90	Scapular fractures (ORIF in local hospital)	Latarjet, Revision ORIF
7	F	69	Left	30	Fractures of Proximal humerus, Bankart lesions	Latarjet, ORIF
8	M	50	Right	32	Osseous Bankart, Hill‐Sachs lesion	Latarjet
9	F	75	Left	21	Glenoid fracture, Hill‐Sachs lesions, Coracoid fracture	Bristow, ORIF
10	F	74	Left	30	Bankart lesion, GT fracture	Latarjet, open reduction and suture fixation
11	F	68	Right	240	GT fracture, osseous Bankart lesion	Bristow, ORIF
12	F	58	Left	24	Glenoid fracture	Coracoid osteotomy and fixed in situ, ORIF
13	M	49	Right	66	Glenoid and acromion fracture, Hill‐Sachs lesion, RCT	Coracoid osteotomy and fixed in situ, ORIF, RCT repair
14	F	66	Right	90	GT Fracture, osseous Bankart, Hill‐Sachs lesion	Bristow, ORIF
15	F	70	Right	28	Osseous Bankart, Hill‐Sachs lesions	Coracoid osteotomy and fixed in situ, ORIF
16	F	62	Left	25	Bankart lesion, Coracoid fracture	Repair of Bankart lesion, ORIF of coracoid fracture
17	F	67	Left	240	Bilateral Osseous Bankart, Hill‐Sachs lesions	Latarjet, Iliac crest bone graft
			Right	60		Bristow
18	F	65	Right	60	Proximal humeral fracture, Hill‐Sachs lesion	Bristow, ORIF, Remplissage

GT, Great tuberosity; RCT, Rotator cuff tear; ORIF, open reduction and internal fixation.

### *Surgical Methods*

#### *Anesthesia and Position*

The patient was in beach chair position with the arm draped free under general anesthesia and a deltopectoral approach was used.

#### *Coracoid Osteotomy*

**Fig 2:**
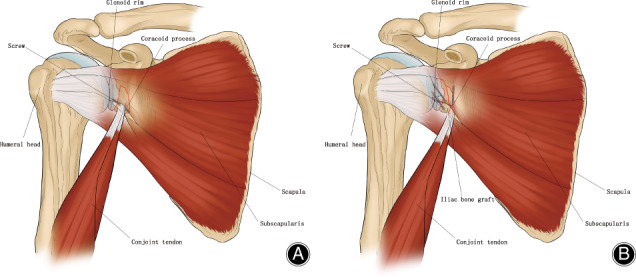
The No.17 patient's surgical diagrams. (A) The surgical diagram showing Bristow procedure in right shoulder. (B) The surgical diagram showing Latarjet procedure and iliac graft block in left shoulder.

The coracoid process was identified and released from the coracoacromial ligament laterally and the pectoralis minor medially. The coracoid was osteotomized at the base and the bone block size was almost 1.5–2.5 cm (Fig. 2).

#### *Capsule Release and Shoulder Reduction*

**Fig 3:**
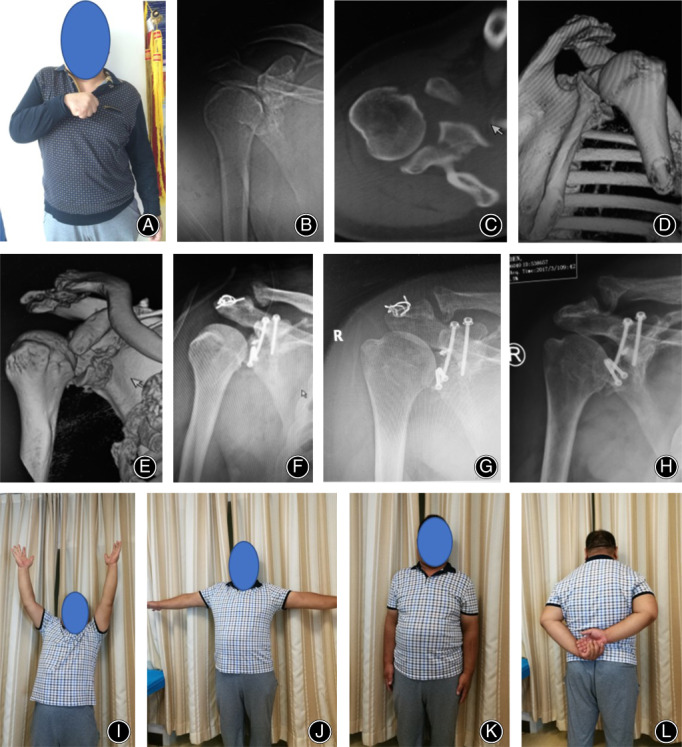
A 49 years old man with glenoid and acromion fractures result to right chronic anterior shoulder subluxation more than 60 days (No.13 patient). (A): This male patient complained severe pain and limited activity. (B): Preoperative anteroposterior radiograph showing a chronic anterior shoulder subluxation. (C‐E): Preoperative CT scans showing chronic anterior subluxation with glenoid and acromion fractures. (F): Anteroposterior radiograph postoperatively showing right shoulder reduction with ORIF of glenoid and acromion factures, coracoid graft was fixed back to scapula with two screws. (G): Anteroposterior radiograph showing right shoulder reduction 1 year postoperatively before the wire tension bend in acromion removing. (H): Anteroposterior radiograph showing right shoulder after the wire tension bend removing. (I‐L): The right shoulder function of this patient basically returned to normal 1 years after surgery.

The rotator interval was opened thoroughly by excising the coracohumeral ligament and the surrounding scar tissue, then the superior and posterior intra‐articular capsular was released underneath the supraspinatus and the infraspinatus. The interval between the humeral head and the anterior aspect of the glenoid was cleared, the dislocated humeral head was gently levered and disengaged from the glenoid rim through the rotator interval with the subscapularis intact and the reduction was achieved with lateral traction and internal rotation. If the reduction was difficult to achieve through the rotator interval, an L‐shaped tenotomy of the subscapularis (the upper two‐thirds was taken down) was performed. After the dislocated humeral head was reduced, open reduction and screw fixation was applied if the glenoid fracture existed (Fig. 3).

#### *Assessment of Shoulder Stability, ±Bristow–Latarjet* *Procedure*

**Fig 4:**
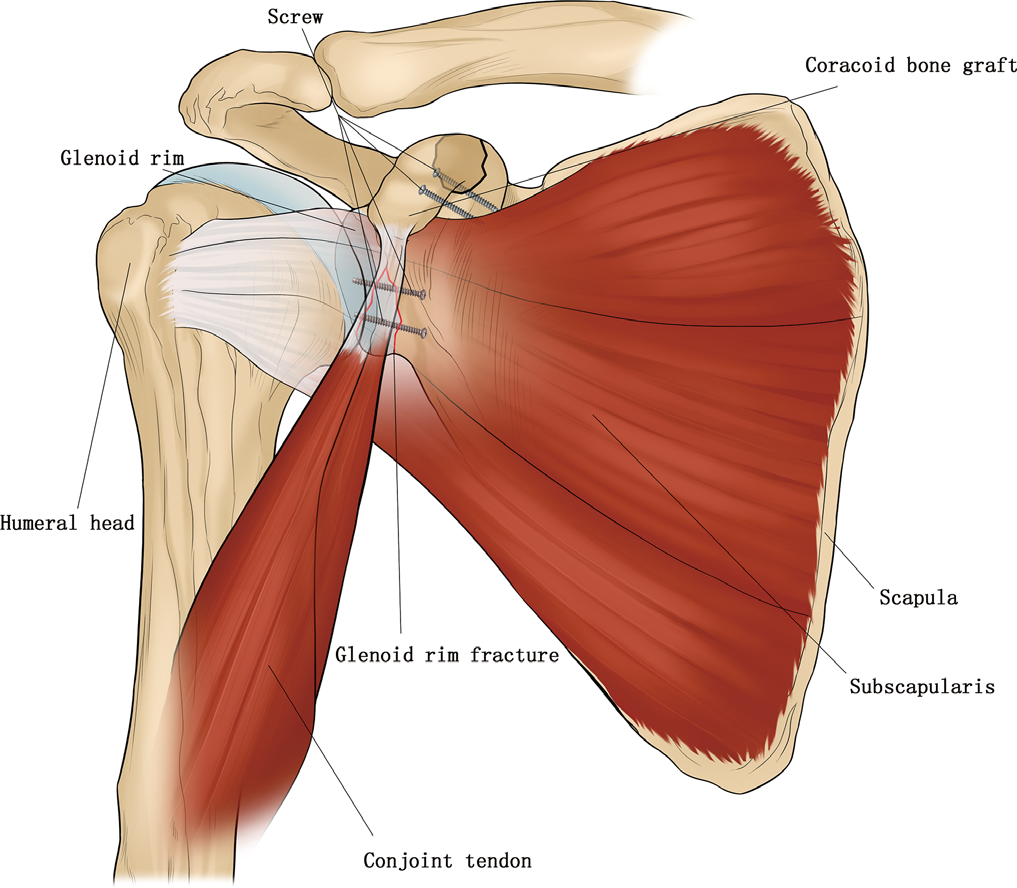
The No.13 patient's surgical diagrams showing open reduction and internal fixation of glenoid and acromion fracture, the coracoid osteotomy and fixed in situ with two screws.

If the shoulder was stable enough, the coracoid bone graft was fixed back to the base of the coracoid with two screws (Fig. 4). If the shoulder was unstable, a Bristow or Latarjet^18^ procedure was performed, the coracoid bone graft with conjoined tendon was fixed to the anterior aspect of the glenoid neck, and the upper portion of the subscapularis was repaired back to the lesser tuberosity (Fig. 1). The displaced osseous Bankart fragments were excised before the coracoid fixation and the glenoid rim injury was repaired with anchors.

#### *Remplissage Technique*

**Fig 5:**
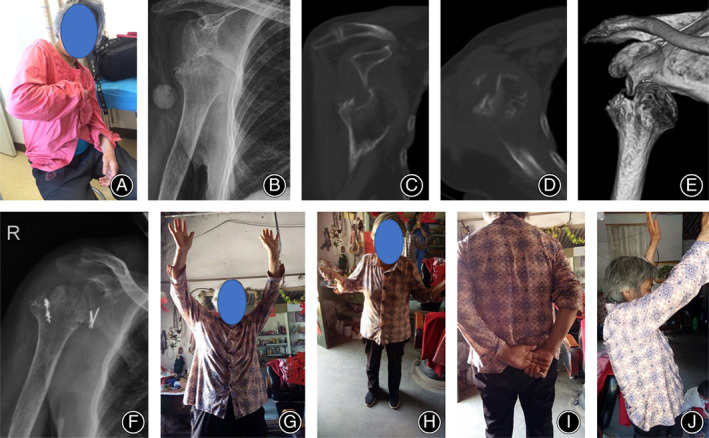
A 69 years old woman with right chronic anterior shoulder dislocation more than 2 months (No.4 patient). (A): This female patient complained severe pain and limited activity. (B): Preoperative anteroposterior radiograph showing a chronic anterior dislocation. (C‐E): Preoperative CT scans showing chronic anterior dislocation with large Hill‐Sachs lesion. (F): Anteroposterior radiograph 3 months postoperatively showing right shoulder reduction with Latarjet procedure and remplissage technique. (G‐J): The right shoulder function of this patient basically returned to normal 2 years after surgery.

**Fig 6:**
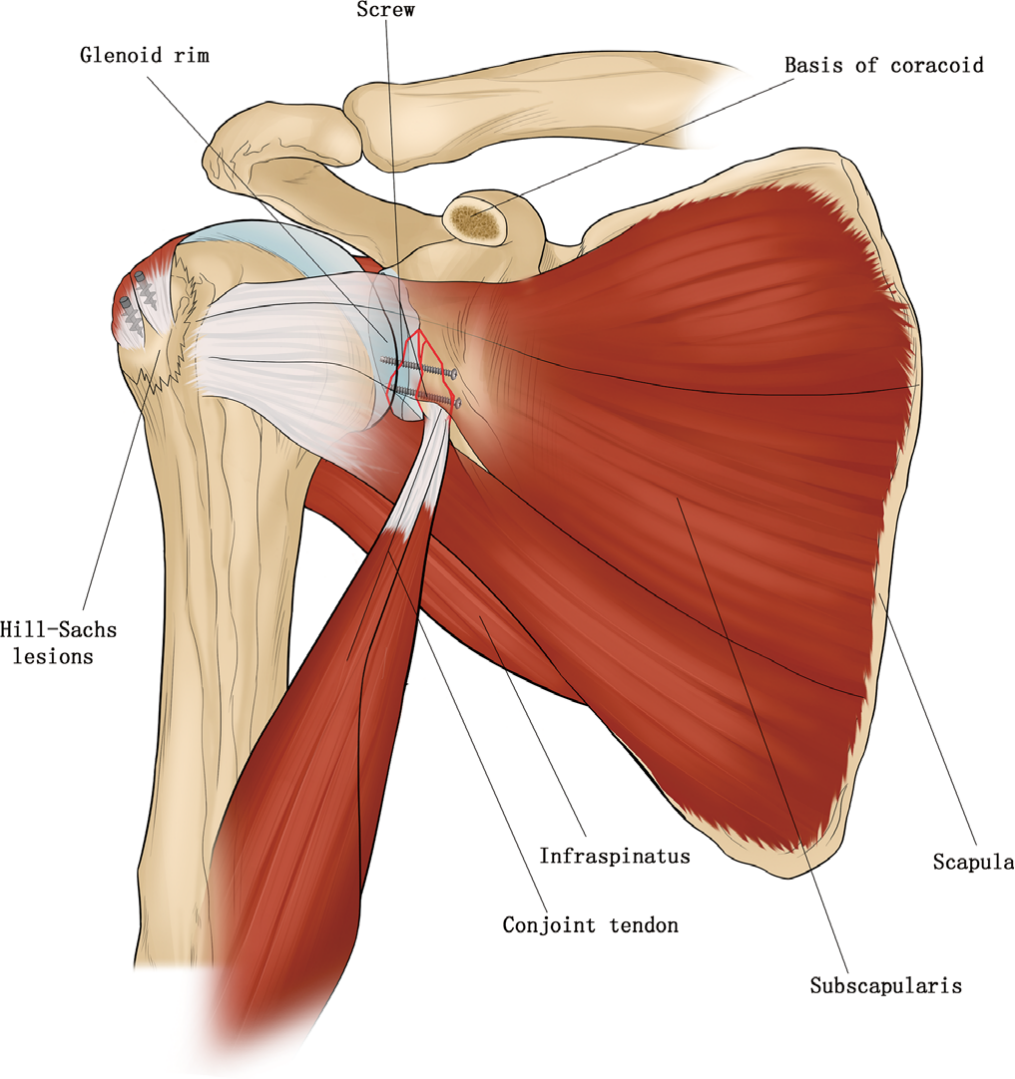
The No.4 patient's surgical diagrams showing Latarjet procedure with remplissage technique.

According to the results of the preoperative three‐dimensional (3D) computed tomography (CT) scans, the proportion of Hill–Sachs lesions accounted for 20%–40% of the humeral head in two patients, and remplissage techniques were applied in these two cases in which the posterior articular capsule and the infraspinatus tendon were filled into the lesions of the humeral heads with anchors (Figs 5 and 6). The proportion was less than 20% of the humeral head in 10 patients, and conservative treatments were applied.

#### *Treatment of Concomitant Injuries*

All concomitant rotator cuff tears were completely repaired with anchors by suture bridge technique. The fracture of great tuberosity was fixed with cannulated screws or anchor suture. The proximal humeral fractures were open reduction and internal fixation (ORIF) with PHILOS (Proximal Humeral Internal Locking Osteosynthesis) plates (Synthes, Stratec Medical ltd, Mezzovico Switzerland) plates. The acromion fracture was ORIF with tension band wires (Fig. 3). One patient underwent ORIF with two reconstructive plates for scapular fractures in another hospital 70 days ago, but his shoulder was still in anterior subluxation position because of glenoid retroversion. The revision surgery was applied for correction of the glenoid retroversion, then followed by the Latarjet procedure.

### *Postoperative Management*

The shoulder was placed in a sling for 6 weeks postoperatively. Active exercise was begun gradually after 6 weeks and strengthening exercise was initiated at 3 months postoperative.

### *Observation Indicators*

#### *The Operation Time*

The operation time was recorded from the beginning of skin incision until surgical closure, which could reflect the proficiency of the operators for these surgical techniques.

#### *The Amount of Operative Blood Loss*

The amount of operative blood loss was the sum of the amount of blood from the suction device and the amount of blood on the gauze.

### *Radiographic Evaluation*

Dislocations were diagnosed on anteroposterior, lateral, and axillary view radiographs and a definite diagnosis was made with a CT scan with 3D reconstruction. The glenoid bone defect^19^ was evaluated on the 3D CT scans. Magnetic resonance imaging (MRI) was made to observe rotator cuff and surrounding soft tissues in all involved shoulders.

### *Follow‐up and Clinical Evaluation*

The patients were followed up at the time of 3 weeks, 6 weeks, 3 months, 6 months, and 12 months postoperatively and at least one time each year. The anteroposterior, lateral, and axillary views of radiographs were adopted to evaluate the position of the humeral head and screws. Whether the implant was stable, whether the shoulder was dislocated, and whether infection was observed.

#### *Visual Analogue Scale (VAS)*

The degree of shoulder joint pain was evaluated by the VAS score. The degree of shoulder pain was evaluated at 3 weeks, 6 weeks, 3 months, 6 months, and 12 months after operation. According to the VAS system, the degree of shoulder joint pain was evaluated in all patients when patients were active. The method involves using the visual analogue graduated scale. One side of the graduated scale was turned back to the patient and the patient was asked to mark the appropriate position on the graduated scale that represents the degree of pain. The score was evaluated according to the patient's mark. The score criteria were as follows: no pain: 0; mild pain, tolerable, not affecting sleep: 1 to 3; moderate pain, mild affecting sleep, still tolerable: 4 to 6; severe pain, unbearable pain, pain resulting in inability to sleep or waking up from sleep: 7 to 10.

#### *Range of Motion (ROM)*

The active shoulder range of motion was evaluated. The ROM of the shoulder included internal rotation, external rotation, forward elevation, and abduction degrees and was evaluated at 3 weeks, 6 weeks, 3 months, 6 months, and 12 months after operation. The degree of internal rotation was evaluated as the highest level that the patient could reach behind the back with the thumb. The forward elevation, external rotation, and abduction degree with the arm at the side were measured with a goniometer.

#### *American Shoulder and Elbow Surgeons (ASES) Score*

The American Shoulder and Elbow Surgeons (ASES) standardized shoulder assessment form contains a patient self‐report section and a section used by medical professionals to record physical examination findings to assess patients with shoulder pathologies. The patient self‐report section consists of two dimensions: pain and activities of daily living. The pain score is calculated from the single pain question and the function scores from the sum of the 10 questions addressing function. The pain score and function composite score are weighted equally and combined for a total score out of a possible 100 points and the higher scores indicate better outcomes. The ASES scores were evaluated at 3 weeks, 6 weeks, 3 months, 6 months, and 12 months after operation.

#### *University of California Los Angeles (UCLA) Score*

The UCLA score is a combined objective and subjective survey that requires completion by both the doctor and patient. It has five sub‐scales made up of: active forward elevation and strength (physician reported), pain, satisfaction, and function (patient reported). A maximum score of 35 is possible with higher scores indicating better outcomes. The UCLA scores were evaluated at 3 weeks, 6 weeks, 3 months, 6 months, and 12 months after operation. All parameters were adopted for functional evaluation by an independent surgeon who was not in the surgical team preoperatively and at the different periods of follow‐up.

#### *Modified Samilson and Prieto Classification System*

According to the modified Samilson and Prieto classification system^20^, the severity of glenohumeral osteoarthritis was evaluated at the time of the last follow‐up: grade 0 was normal; grade 1 was defined as inferior humeral or glenoid osteophytes or both, measuring <3 mm in height; grade 2 was inferior humeral or glenoid osteophytes, or both, between 3 mm and 7 mm in height, with slight glenohumeral joint irregularity; and grade 3 was inferior humeral or glenoid osteophytes, or both, >7 mm in height, with narrowing of the glenohumeral joint and sclerosis.

### *Statistical Analysis*

Statistical software IBM 20.0 (International Business Machines Corporation, Armonk, New York, USA) was used for statistical analysis. Parametric data, such as VAS, ASES and UCLA scores, were described as mean ± SEM and compared using Student *t‐*tests. Proportional values were compared using χ^2^‐analysis or Fisher exact test where applicable. For each test, a *P* value < 0.05 was defined as significant.

## Results

### *Surgical Outcomes*

The mean operation time was 156.5 ± 5.5 min (range, 120–210 min). For patients with bilateral dislocations, one side of the dislocated shoulders underwent operation first, and the other side was operated one week later. The mean amount of operative blood loss was 340.0 ± 24.5 mL, (range, 200–600 mL). Twenty shoulders of 18 patients with chronic anterior dislocation were treated with open reduction and coracoid osteotomy with or without Bristow–Latarjet procedures. Latarjet procedures were applied in nine shoulders (Fig. 6), Bristow procedures were in seven shoulders (Fig. 2), the coracoid bone grafts were fixed back to the base of the coracoid process in three shoulders (Fig. 4), and coracoid fracture was reduced and fixed with two cortical screws in one patient. The fractures of the great tuberosity were fixed with screws or anchors in six shoulders and the proximal humeral fractures with PHILOS plates in two shoulders. The scapular or glenoid fractures were fixed with reconstructive plates or screws in four shoulders. The rotator cuff tears were repaired with anchors by suture bridge techniques in five shoulders.

### *Follow‐up*

All patients were followed up with clinical examination and radiographs. The mean follow‐up time was 15.2 ± 4.3 months (range, 12–24 months). During the period of follow‐up, subluxation after surgical procedure was found and confirmed on CT scans in one patient and the patient refused to undergo revision surgery again. According to the Samilson and Prieto classification system, 16 shoulders were assessed as grade 0, three shoulders were grade 1, one shoulder was grade 2. Sixteen shoulders of 14 patients treated with Bristow–Latarjet procedures were followed up and received functional evaluation.

#### *Forward Elevation*

At the time of follow‐up, the mean degree of forward elevation was improved from 67.5° ± 2.33° preoperatively to 96.88° ± 2.85° at 3 months postoperatively (*t* = 7.99, *P* < 0.001), and to 115.0° ± 3.65° at 12 months postoperatively (*t* = 10.97, *P* < 0.001). The degree of forward elevation was improved by 43.53% at 3 months and by 70.37% at 12 months postoperatively compared with the degree preoperatively, respectively.

### *Internal and External Rotation*

The mean internal rotation had increased from the buttock to L_2_ at the last follow‐up (*P* = 0.06). The mean degree of external rotation was not different from the mean degree at 3 weeks (*t* = 0.21, *P* = 0.83) or 6 weeks (*t* = 0.74, *P* = 0.46) postoperatively, but it was improved from 9.4° ± 2.14° preoperatively to 30.6° ± 3.59° at 1 year postoperatively (*t* = 5.09, *P* < 0.001). The degree of external rotation was improved by 225.53% at 12 months postoperatively compared with the degree preoperatively.

### *Abduction*

The mean degree of abduction had increased from 48.8 ^o^ ± 2.40^o^ to 58.1^o^ ± 1.88^o^ at 3 weeks after operation (*t* = 3.08, *P* = 0.004), and to 110.5° ±° 4.33° at 1 year postoperatively (*t* = 12.51, *P* < 0.001) (Table 2); The degree of abduction was improved by 19.06% at 3 weeks postoperatively and by 126.43% at 1 year postoperatively compared with the degree preoperatively, respectively. The mean active ROM of the involved shoulders failed to return to the active ROM of the healthy shoulders.

**TABLE 2:** Postoperative outcome of patients with Bristow‐Latarjet procedures

Evaluation items	Preoperative	3 weeks postoperatively	6 weeks postoperatively	3 months postoperatively	6 months postoperatively	12 months postoperatively
VAS	6.0 ± 0.22	5.3 ± 0.24*	4.3 ± 0.19*	3.3 ± 0.21*	1.9 ± 0.25*	1.5 ± 0.26*
Forward elevation (deg)	67.5° ± 2.33°	76.0° ± 2.47°*	85.0° ± 2.89°*	96.9° ± 2.85°*	108.8° ± 3.01°*	115.0° ± 3.65°*
External rotation (deg)	9.4° ± 2.14°	8.8° ± 2.02°	11.9° ± 2.62°	17.8° ± 2.46°*	26.3° ± 2.87°*	30.6^o^ ± 3.59°*
Abduction (deg)	48.8° ± 2.40^o^	58.1° ± 1.88^o^ *	73.1° ± 2.70^o^ *	90.6° ± 3.71^o^ *	103.8° ± 4.17^o^ *	110.6° ± 4.33^o^ *
ASES	39.1 ± 2.11	40.8 ± 2.11	50.8 ± 2.23*	65.5 ± 2.03*	75.2 ± 2.04*	83.4 ± 1.96*
UCLA	10.2 ± 0.58	11.6 ± 0.69	14.0 ± 0.78*	17.1 ± 1.05*	20.5.2 ± 1.32*	22.56 ± 1.49*

VAS, Visual Analogue Scale; deg, degree; ASES, American Shoulder and Elbow surgeons score; UCLA, University of California Los Angeles score.

* Means significant (*P* value <0.05), the Parameters in different follow‐up period after operation were compared with those preoperatively.

### *Visual Analogue Score (VAS)*

The mean VAS had significantly decreased from 6.0 ± 0.22 points preoperatively to 5.3 ± 0.24 at 3 weeks postoperatively (*t* = 2.11, *P* = 0.04), and to 1.5 ± 0.26 points at 12 months follow‐up (*t* = 13.17, *P* < 0.001) (Table 2). The VAS was reduced by 11.67% at 3 weeks postoperatively and by 75.0% at 1 year postoperatively compared with the preoperative VAS, respectively.

### *American Shoulder and Elbow Surgeons (ASES) Score*

The mean ASES score had improved from 39.1 ± 2.11 points to 83.4 ± 1.96 points at the 12 months follow‐up (*t* = 15.40, *P* < 0.001), the ASES score was improved by 113.30% at 12 months postoperatively compared with the preoperative ASES score. But the score was not significantly improved at 3 weeks postoperatively (*t* = 0.57, *P =* 0.5762), the score was significantly improved from 6 weeks after operation (Table 2).

### *University of California Los Angeles (UCLA) Score*

The mean UCLA score had significantly improved from 10.2 ± 0.58 points to 14.0 ± 0.78 points at 6 weeks follow‐up (*t* = 3.93, *P* = 0.005) and to 22.56 ± 1.49 at the 12 months postoperatively (*t* = 7.75, *P* < 0.001). The UCLA score was reduced by 37.25% at 6 weeks postoperatively and by 121.18% at 12 months postoperatively. But it was not significantly improved at 3 weeks postoperatively (*t* = 1.53, *P*= 0.14) (Table 2).

### *Complications*

No procedure‐related death or vascular injury was identified in all cases. An axillary nerve injury was suspected because of numbness of lateral skin of the shoulder in one patient postoperatively, and they recovered 1 month after surgery. No incision‐related superficial or deep tissue infection was found in these cases. No osteonecrosis of humeral head or iatrogenic neurovascular injuries as well as fractures were found in this study.

## Discussion

Anterior shoulder dislocations represent the most common of all dislocations. The chronic anterior shoulder dislocation was usually defined as the duration of dislocation more than 3 weeks old^21^. But Goga *et al* ^2^. presented a new classification for shoulder dislocation which stated that dislocations more than 1 week old should be termed chronic. Chronic dislocations should be further classified into early (>1 week but <3 week old), late (>3 week but <12 week old), and ancient ( >12 week old). In these series cases, the mean duration between dislocation and surgical procedures was 73.3 ± 14.4 days. All patients who underwent surgical treatment complained of severe pain, limited activity, and declined quality of life. Though very few cases reported that patients with chronic irreducible anterior dislocation were without significant functional deficit^5^, most cases underwent surgical treatment to maintain patients' active lifestyle, including weightlifting and manual work.

There were several published reports about the treatment of chronic anterior shoulder dislocation, and the majority were case reports. The choice of treatment depended on the following factors: the duration of dislocation^2^, dislocation associated with bone and soft tissues injuries, age, dominant hand, functional requirements, and complications of the treatment. Based on the previous research results, conservative treatment was suitable for patients with long‐time dislocation, non‐dominant hand involvement, and low functional requirement. The failure rate of closed reduction was high due to soft tissue obstruction (long head of biceps, intra‐glenoid scar, or posterior capsular contracture) and osseous block (glenoid fractures or engaging Hill–Sachs lesions), meanwhile the danger of an iatrogenic fracture or neurovascular damage was also high^8^, ^22^. The pathophysiological changes of the chronic anterior shoulder dislocation were different from those of recurrent anterior dislocation, which also limited the application of arthroscopy^6^, ^23^, ^24^. In this study, the duration of dislocation was more than 3 weeks in all cases, and all patients underwent open reduction and stabilization of the involved shoulder. There were no iatrogenic fractures and neurovascular injuries or incision‐related complications that occurred in this study.

The optimal surgical treatment method for chronic anterior dislocation is still controversial. Li and Jiang^6^ considered that hemiarthroplasty, even with a concomitant Latarjet procedure, was consistently unsuccessful for chronic anterior dislocation in their cases. Kohan *et al* ^25^. reported their results of reverse total shoulder arthroplasty for 22 patients with dislocation, including 14 early and five late dislocations. Recurrent instability after revision was present in 29% of early and 40% of late dislocators. They concluded that the post‐RTSA instability had two distinct etiologies: (i) instability due to inadequate soft tissue tensioning and/or axillary nerve palsy; and (ii) instability due to impingement or liner failure. Bah *et al* ^24^. compared arthroscopic Bankart with remplissage with open Latarjet procedure for chronic anterior shoulder instability with significant Hill–Sachs lesion. Their conclusions were that the two surgical methods were reliable and safe procedures associated with low and similar recurrence rates. However, loss of external rotation and residual pain were significantly more common with the combined Bankart–remplissage procedure. For treatment of recurrent anterior shoulder dislocation with a marked glenoid osseous defect, an open Latarjet procedure was more effective compared with Bankart repair^18^, ^26^. Li and Jiang^6^ also considered that the Latarjet procedure for the treatment of chronic locked anterior shoulder dislocation could be successful if the shoulder reduction can be performed without requiring a subscapularis tenotomy. The Latarjet procedure performed with subscapularis tenotomy and repair was found to have a high or persistent glenohumeral instability, re‐dislocation or subluxation; limited shoulder range of motion; and early onset of glenohumeral osteoarthritis. In this study, the osteotomy with or without Bristow or Latarjet procedure with subscapularis tenotomy was performed and one re‐dislocation was found in the last follow‐up. The function of the dislocated shoulder at the last follow‐up was better than its function preoperatively, but worse than the function of the contralateral side. The insignificant improvement of external rotation at 3 and 6 weeks after operation might be due to the use of shoulder abduction braces. For patients, functional exercise of postoperative external rotation was the most difficult relative to other angles. In the cases chronic anterior shoulder dislocation, it was difficult to reduce the humeral head because of long‐term contracture and scarring adhesion of peripheral soft tissue if the subscapularis was not tenotomy, and the risk of iatrogenic injuries was high. Our results were not the same as that reported by Y Li, maybe because the duration of anterior locked dislocation in their cases was more than 3 months and the mean follow‐up was 31.6 months.

In our case series, the concomitant injuries were common, including Hill–Sachs and Bankart lesions, massive glenoid bone loss, rotator cuff tear, scapular or glenoid fracture, and proximal humeral fracture. It was difficult to perform all surgical procedures through the conventional approaches, such as deltopectoral approach. The coracoid osteotomy was our routine procedure for chronic anterior dislocation. The advantages of coracoid osteotomy were as follows: (i) it could enlarge the exposure of surgical field by relieving the tension of the conjoined tendon and facilitate to release the contracture of soft tissue and reduce the dislocated shoulder; (ii) it was easy to apply the Bristow or Latarjet procedure if the dislocated shoulder was not stable after reduction; otherwise, the coracoid bone graft was fixed back to the base of the coracoid process. In our study, the coracoid grafts were fixed back to the base in four of 20 shoulders, the Bristow or Latarjet procedures were applied in 16 shoulders and the functional outcomes were significantly improved at the last follow‐up.

According to our experiences, there were several key points for coracoid osteotomy for chronic anterior dislocation: (i) the procedure (Bristow or Latarjet) used depended on the size of coracoid process – coracoid fractures were found in three of 20 shoulders and coracoid osteotomy was not necessary; (ii) when separating the conjoint tendons after coracoid osteotomy, we were careful to avoid damage of the musculocutaneous nerve; (iii) the location of the coracoid graft should be confirmed repeatedly to avoid being higher than articular surface; (iv) pay attention to avoid using any instrument to poke into humeral head results in iatrogenic fractures because different degrees of osteoporosis occurring from long‐term dislocation; (v) pay attention to treat the concomitant injuries, such as rotator cuff tear, humeral or scapular fractures; (vi) early scientific and reasonable rehabilitation exercise after surgery was helpful for the recovery of shoulder function.

There were several limitations in this study. First, this study was a retrospective clinical case analysis without the control group, and not a prospective study. Second, the sample size was not large enough because the chronic anterior shoulder dislocation is a very rare condition. Lastly, our clinical results are not accurate because our follow‐up time was not long enough.

### *Conclusions*

In conclusion, coracoid osteotomy with or without Bristow–Latarjet procedure yielded an acceptable clinical result in this study. This method has the advantages of enlarging the exposure of surgical field, assisting reduction of shoulder, and convenient conversion to Bristow–Latarjet procedure. The concomitant injuries treated properly during the operation and early postoperative rehabilitation exercise were important for improving the curative effect of surgery. This study shows that coracoid osteotomy with or without Bristow–Latarjet procedure is an efficient and reliable method for treatment of chronic anterior shoulder dislocation.
